# The rise of scientific machine learning: a perspective on combining mechanistic modelling with machine learning for systems biology

**DOI:** 10.3389/fsysb.2024.1407994

**Published:** 2024-08-02

**Authors:** Ben Noordijk, Monica L. Garcia Gomez, Kirsten H. W. J. ten Tusscher, Dick de Ridder, Aalt D. J. van Dijk, Robert W. Smith

**Affiliations:** ^1^ Bioinformatics Group, Wageningen University and Research, Wageningen, Netherlands; ^2^ CropXR Institute, Utrecht, Netherlands; ^3^ Experimental and Computational Plant Development, Institute of Environmental Biology; Theoretical Biology, Institute of Biodynamics and Biocomplexity, Department of Biology, Utrecht University, Utrecht, Netherlands; ^4^ Biosystems Data Analysis, Swammerdam Institute for Life Sciences, University of Amsterdam, Amsterdam, Netherlands; ^5^ Laboratory of Systems and Synthetic Biology, Wageningen University and Research, Wageningen, Netherlands

**Keywords:** machine learning, mechanistic models, scientific machine learning (SciML), ordinary differential equations, system identification, parameter estimation, biology-informed neural network (BINN)

## Abstract

Both machine learning and mechanistic modelling approaches have been used independently with great success in systems biology. Machine learning excels in deriving statistical relationships and quantitative prediction from data, while mechanistic modelling is a powerful approach to capture knowledge and infer causal mechanisms underpinning biological phenomena. Importantly, the strengths of one are the weaknesses of the other, which suggests that substantial gains can be made by combining machine learning with mechanistic modelling, a field referred to as Scientific Machine Learning (SciML). In this review we discuss recent advances in combining these two approaches for systems biology, and point out future avenues for its application in the biological sciences.

## 1 Introduction

Classically, systems biology has primarily focused on the use of dynamic mechanistic models to elucidate the underpinnings of natural phenomena. Popular model formalisms applied include ordinary and partial differential equations (ODEs and PDEs, respectively), Boolean networks, Petri nets, cellular automata, individual-based models, and combinations of these. Properties of mechanistic models—including the type of equation or rules, initial conditions, or parameter values—depend on the field, question of interest, and expertise of the researchers involved and are often determined or constrained by the limited availability and quality of experimental data. While classic, low-dimensional models can fit a range of concentration-, time-, and space-dependent datasets (Michaelis and Menten, 1913; Lotka, 1920; Volterra, 1926; Hodgkin and Huxley, 1952), for larger, high-dimensional biological systems such models can be difficult to construct due to the so-called curse of dimensionality (Bellman, 1957): as many variables and hence model parameters are necessary to describe a high-dimensional system, it is virtually impossible to generate sufficient experimental measurements to properly estimate these parameters. Only if many existing parameters are known *a priori* (e.g., reaction rates from experimental measurements), they can be used to construct a quantitative mechanistic model that overcomes the curse of dimensionality (Karr et al., 2012). Alternatively, coarser models such as Flux Balance Analysis and Boolean models are typically applied to large metabolic or regulatory networks, as their assumptions lead to simpler models (Xiao, 2009; Orth et al., 2010). Mechanistic models have been indispensable tools to test if our current understanding of biology is necessary and sufficient to describe experimental data, all while having interpretable inner workings. Nevertheless, a gap exists whereby high-throughput time- or space-dependent data is not yet readily used to construct detailed, large mechanistic models.

More recently, state-of-the art machine learning (ML) algorithms have been developed and applied to the increasing wealth of biological data. Since these are data-driven methods that are built to infer patterns from large, high-dimensional datasets, they have enabled high accuracy in applications such as protein structure and function prediction (Jumper et al., 2021; Kulmanov et al., 2018), single-cell transcriptomics modelling (Lopez et al., 2018), and more (see Baker et al., 2018; Sapoval et al., 2022). However, many of these ML methods have limited biological interpretability, and do not elucidate underlying biological mechanisms in the way that mechanistic models can.

Given their complementary strengths and weaknesses, integration between ML and mechanistic models, also called SciML, is a promising new field, which has already gained popularity in scientific disciplines such as engineering (Willard et al., 2022), crop modelling (Maestrini et al., 2022), and physics (Karniadakis et al., 2021). Indeed, there is a great interest in combining these two approaches and their application in diverse fields (Legaard et al., 2023; Tong et al., 2020; von Rueden et al., 2021). In this review, we discuss the latest advances in combining ML and mechanistic modelling approaches—particularly in the form of ODEs or PDEs—applied to systems biology. Notably, while similar reviews for fields like biomedical multiscale models exist (Alber et al., 2019), and reviews such as Gazestani and Lewis (2019) concentrate solely on deep learning—a subset of ML—our focus is on innovative approaches in merging biological knowledge with various ML approaches within the systems biology domain. Here, we aim to provide a perspective on the use of SciML for the study of biological systems, and thus we do not explicitly focus on performing the modelling in practice. For more information on SciML-related software packages and best practices, please refer to the Supplementary Material.

We first describe methods leveraging prior biological knowledge or mechanistic models to augment the interpretability and accuracy of ML models. Subsequently, we explore how ML techniques can contribute to the development and simulation of mechanistic models. Next, we review models that intrinsically merge mechanistic models with ML, and the synergy this provides. Finally, we provide a perspective on potential new avenues for integration of ML and mechanistic models. A brief overview of all categories of models that we discuss is given in Table 1, where we highlight what mechanistic model and ML building blocks they are built of, and for what goal they are integrated.

**TABLE 1:** Overview of the SciML approaches covered in this review, the models they merge, and the goal of integration (NN, neural network, MM, mechanistic model, ML, machine learning, ODE, ordinary differential equation).

Section	Name	Starting point	Combine with	Goal
2.1	Constraining ML model structure	Standard fully connected NN	Dataset of (predicted) biological interactions, only connect nodes in NN if there is evidence for an interaction	Make nodes and edges take on meaning; increase interpretability
2.2	Mechanistic model simulations as input for ML	Existing MM	NN to make predictions based on MM output	Perform task that MM could not do in isolation
3.1	Selecting from a library of candidate terms	Terms from which ODEs could be constructed	ML to select key terms from the library	Identify ODEs that fit dataset using a small number of candidate terms
3.2	Finding hidden mechanisms	ODEs with some terms (i.e., mechanisms) already known	NN to fit unknown terms	ODE model with increased performance; potentially information about what terms should be added to the ODEs
3.3	No candidate terms are known	ODEs missing terms that are needed to explain rate of change	NN that predicts the rate of change of each element (e.g., gene), based on all other elements in the system	Accurate, but hard to interpret method to predict temporal patterns
3.4	NN to enhance model simulations	Parameterised ODEs	NN that predicts the solution of the ODEs	Faster solving of the ODE system
4.1	ML to aid in fitting sparse, noisy data	ODEs that should be fit to noisy and/or sparse data	NN to interpolate the data while adhering to the limits that the ODEs provide	Interpolate data (without overfitting) for finding parameters of ODEs
4.2	Parametrisation of metabolic systems	High-dimensional system of ODEs with yet unknown parameters	NN that predicts a set of parameters, and NN that can classify if parameters are good or not	Find parameters for large system of ODEs that make it consistent with experimental data

## 2 Combining ML with prior knowledge

### 2.1 Constraining ML model structure

Machine learning is concerned with computational methods that learn (i.e., are trained) to perform a certain task based on example data. A wide range of methods are available, each differing primarily in the assumptions they impose on a problem. This results in a trade-off between the model's complexity and its ability to learn any given problem, known as the bias-variance trade-off (Geman et al., 1992). As a major subfield of ML, neural networks (NNs, more recently called deep learning, DL) consist of simple functions (“units” or “nodes”) that calculate a weighted combination of their inputs and then apply a non-linear transformation to produce an output. By combining several layers of such units, given a dataset of examples of input x and desired output y , sufficiently large NNs can in principle be trained to approximate any function (Hornik et al., 1989)

$$\hat{y}=\text{NN}\left(x,w,b\right)$$ (1)

where w and b represent the internal weights and biases of the NN, respectively. For readability, subsequent equations will omit explicit mention of these parameters.

NNs have shown great potential in systems biology (Sapoval et al., 2022) to, for example, relate multi-omics data to drug response (Sharifi-Noghabi et al., 2019). Nevertheless, the broad deployment and practical utility of NNs is still limited by a number of factors. First, NNs can be hard to generalise to different biological contexts as they easily overfit the specific training data available. Second, as highly parameterised universal approximation methods, NNs suffer from a lack of interpretability. Therefore, it makes sense to inform NNs with existing biological knowledge to constrain their complexity, a task for which NNs are well-suited. Conventionally, such approaches start from an existing NN architecture (e.g., a multi-layer perceptron, MLP, or a recurrent NN, RNN) and limit some of its internal connections based on biological data or prior knowledge, thus reducing the number of parameters to be estimated. In some cases, this allows certain elements of the NN to take on a mechanistic meaning, which “opens up the black box.” Here we discuss methods where NN performance and/or interpretability has been aided by inclusion of established biological insights.

A first way to enforce biological prior knowledge is by creating a sparsely connected MLP, where each node represents a biological entity (e.g., a gene, protein complex, or full cell organelle) and nodes are only connected if they are known to interact based on experimental or computational biological evidence (Elmarakeby et al., 2021). Such a sparse MLP has been applied to cell growth models, where connections were informed by Gene Ontology (GO) terms (Ma et al., 2018) and to modelling signalling and transcriptional regulation, where each connection is based on known interactions between genes, proteins, and their pathway membership (Fortelny and Bock, 2020; Hartman et al., 2023). Overall, these studies find that such biologically-constrained MLPs outperform existing predictive models, suffer less from overfitting compared to their fully connected counterparts, and allow for meaningful biological interpretability. However, there is no agreed upon best method yet to extract biological insights from these sparse MLPs.

MLPs are not the only NN architecture that can be used as a blueprint for biology-informed ML. For example, in a recurrent neural network (RNN), the matrix governing the calculation of the hidden state from the previous time point’s hidden state can be likened to an interaction matrix (graph) between molecules in a signalling network (Nilsson et al., 2022). Therefore, this matrix can be constrained to only include known interactions, which prevents overfitting, and enables genome-scale modelling of intracellular signalling. Moreover, this matrix can be further constrained by existing knowledge of dynamical systems, e.g., by restraining the system’s largest eigenvalue to be smaller than one, as this ensures that the RNN always converges to a steady state or equilibrium. Other architectures, such as convolutional neural networks (CNN), have also been constrained with prior knowledge in fields such as physics (Zhang Z. et al., 2023). However, in the field of systems biology we were unable to find examples of such applications yet, even though CNNs could be used to study, e.g., spatial cell-cell interactions.

Overall, this highlights the potential for constructing biologically-constrained NNs by starting with existing NN architectures that effectively align with the structure of the biological problem being addressed. Nevertheless, not all prior biological knowledge naturally lends itself to this, and the most insightful way to extract meaning from the internal workings of an NN remains to be elucidated.

### 2.2 Mechanistic model simulations as input

An alternative way to make use of biological knowledge is to use the output of mechanistic models (defined more in depth in Section 3) as “input” to an ML model (Gelbach et al., 2022; Myers et al., 2023). Note that this should be distinguished from “integrated models,” where part of the system is modelled using ODEs and another part using ML; here, we focus on cases where multiple ODE simulations are performed to generate data to train the ML model.

One classic approach is so-called simulation-based inference, which refers to a suite of techniques for inferring model parameters when the likelihood function is not tractable (Cranmer et al., 2020). A likelihood function quantifies the probability of observing a set of data given a specific set of parameter values in a model. Parameter values can then be optimised by maximising this likelihood. Classical approaches for simulation-based inference include, e.g., approximate Bayesian computation (ABC), where parameters are repeatedly drawn from a prior distribution, a simulation is run with those parameters, and the parameter values are retained as a sample of the posterior distribution if the simulated data is sufficiently close to the observed data. This yields a probability distribution for parameter values given a model structure and a dataset. The approach is case-based, in the sense that for a new set of observations, the entire estimation procedure must be run again.

A second approach is to create a model for the likelihood by estimating the distribution of simulated data with, e.g., kernel density estimation. Compared to ABC, it has the advantage of spreading the costs of the initial investment in simulation across various analyses or parameter estimates: new data points can be evaluated more efficiently. Here, recent developments that use NNs now allow density estimation to scale to high-dimensional data. An example is normalising flows, in which variables described by, e.g., a multivariate Gaussian are transformed through a parameterised invertible transformation. Several such steps can be stacked, and the parameters of the transformations are trained by maximising the likelihood of the observed data. A recent example of such an approach is Bayesflow (Radev et al., 2020), which trains two neural networks on simulated data: i) a summary network, which reduces a set of observations to learned summary statistics (for time-series, typically a long-short-term memory (LSTM) network is used, which is a variant of the above-mentioned RNN); and ii) an inference network, which learns the posterior given these summary statistics. The latter is implemented as a normalising flow. Bayesflow has been used for systems biology problems in Arruda et al. (2023) to consider measurements for different cells or patients, and simulate a heterogeneous cell population using a non-linear mixed-effects model of (single-cell) translation.

An alternative to simulation-based inference is to use transfer learning (Przedborski et al., 2021). This leverages features and representations learned by solving one problem to help solve a related but different problem. After pretraining a model on a large dataset, it can be transferred and fine-tuned for a new task with smaller datasets, accelerating learning and improving performance. This approach is especially useful when labelled data for the target task is limited or expensive to obtain. In the specific example of Przedborski et al. (2021), simulated clinical trial data was obtained from an already calibrated ODE model for immunotherapy, describing time evolution of various cell types based on molecular interactions. Note that this existing model was not directly aimed at distinguishing between patients responding and non-responding to treatment. To do so, an additional classification model was developed. Relevant features for distinguishing response from non-response were selected from the initial conditions and kinetic parameters of the ODE model simulations. These features were then used as inputs to an NN, which was pretrained on the simulated data to classify virtual patients as responders or non-responders. Subsequently, transfer learning was used to fine-tune the model on real clinical data.

Both biologically-constrained MLPs and ODE-input ML have typically been applied to datasets where the final output is static (i.e. a state that does not change). For dynamic outputs, it may be better to start with a mechanistic model and enhance it using ML, as discussed in the next section.

## 3 ML to enhance mechanistic models

Ordinary differential equation (ODE) models are a commonly used framework to model biological dynamical systems. As the affordability and accessibility of many experimental methods have increased, and the scale of data generation has grown dramatically, mechanistic models have become larger (Fröhlich et al., 2018), more detailed, and less abstract. This leads to a need for both new methods for model construction (i.e., identifying the unknown terms in an equation), and for improved numerical algorithms to address the high computational requirements of ODE solving. Here, we discuss four ways in which ML can support the construction and simulation of mechanistic models: i) if potential terms in the ODE are already known and a subset should be selected, ii) if some terms are still unknown, iii) if all candidate terms are unknown, and iv) if ODE solving should be enhanced.

### 3.1 Selecting from a library of candidate terms

The first step of any mechanistic modelling study is to define the equations of the model based on prior knowledge of the biological system. These equations describe the rate at which a variable changes over time and/or space, and how it depends on other variables in the system and parameters/reaction rates. The mathematical notation for such a system generally reads

$$\frac{dx}{dt}=f\left(x,p,t\right)$$ (2)

where dx/dt is the rate of change of species or variables x over time, which is determined by reactions f with parameters (or rate constants) p . These reactions may be influenced by time t . In systems biology, the functions f could represent defined chemical reactions between variables, e.g., conversions between different states or enzyme-catalyzed Michaelis-Menten reactions, that depend on parameters p with clear biological definitions, e.g., transcription, translation, complex formation, (de)phosphorylation, dilution, degradation, and diffusion rates. Consequently, many systems biology models are constructed from the same set of mathematical terms, or building blocks, with a direct biological interpretation (Ingalls, 2013; Klipp et al., 2016).

Another factor to consider is the size of the model, i.e. the number of variables and/or parameters. This is often constrained by the data availability, namely which system species and rates have been measured. In the process of model construction, a key question for the modeller is then whether a model needs to be complete—in the sense that all known variables x need to be contained within the model—or whether a smaller, abstract model is sufficient to explain the available data. This is referred to as *model parsimony* and measures such as the Akaike Information Criterion can be used to compare model structures (Portet, 2020). In practice, this means that systems biologists often search for models with a limited number of “hidden,” or unmeasured, variables to reduce the uncertainty in predictions made for measured variables.

Both considerations above—equation formulation and model size—can be biased by the researchers’ preferences and prior knowledge. To avoid this, ML has recently been applied to construct models based on data in an unbiased manner. For example, Erdem and Birtwistle (2023) utilised ML to infer gene networks from integrated -omics data and used these connections to expand an existing mechanistic model (Erdem et al., 2022; 2023). Alternatively, when a library of potential terms in f is already known, the SINDy (sparse identification of non-linear dynamics) family of symbolic regression methods has been developed to select the most relevant terms from this library (Brunton et al., 2016; Champion et al., 2019; Massonis et al., 2023). The SINDy method (Brunton et al., 2016) rewrites an ODE, as in Eq. 2, into

$$f\left(x\right)=\Theta \left(X\right)\Xi$$ (3)

where Θ(X) is a time-dependent matrix containing a library of candidate mathematical terms for the ODE (e.g., cos(x(t)) , $x^{2}\left(t\right)$ , …), and Ξ is a sparse matrix containing parameters detailing the rates of each associated mathematical term in the equation. To obtain the matrix Ξ from data, we can minimise a loss function

$$L={\left(\frac{dx_{d}}{dt}-\Theta \left(X\right)\Xi \right)}^{2}$$ (4)

where $dx_{d}/dt$ is the numerically approximated time-derivative of time-dependent measurements. When the loss function L approaches zero, the predicted ODEs produce solutions that match the time-dependent measurements of variables. To prevent complex models being obtained, this optimisation problem is solved with sparse regression methods, such that Ξ is a sparse vector containing as many zeros as possible (Brunton et al., 2016). Test cases in the literature encompass a variety of oscillatory systems, including Lorenz attractors, swinging pendulums (which have recently been related to cell cycle models (Dragoi et al., 2024)), spatial patterning, and glycoloysis pathways in yeast. Moreover this SINDy methodology has since been extended to model non-linear dynamics using implicit functions (Kaheman et al., 2020) and to create structurally identifiable models (Massonis et al., 2023). One recent extension of the SINDy method used autoencoder NNs to reduce the dimensionality of data x to a smaller set of “intrinsic coordinates” z , which can be modelled and used to reproduce the observations seen in the larger system (Champion et al., 2019). In this instance the neural network calculates

$$z=\text{NN}\left(x\right)$$ (5)

where |z|<|x| , and dz/dt provides knowledge about the larger system dx/dt . Compared to linear dimension reduction approaches such as principal component analysis or dynamic mode decomposition, this nonlinear approach may lead to poor interpretability of the dynamic variables, but it allows for more complex models to be simplified and analytically explored.

### 3.2 Finding hidden mechanisms

In a second, less constrained, modelling approach, universal ordinary differential equations incorporate NNs into the differential equations themselves. In this case, the mathematical definition of a reaction or relationship between model variables may be unknown, and a neural network is trained to determine the time-dependent rate of change. An example universal ordinary differential equation would then take the form

$$\frac{dx}{dt}=f\left(x\right)+\text{NN}\left(x,t\right)$$ (6)

where f(x) models known relations, whilst NN(x,t) is a time-dependent NN that represents unknown interactions. The equations are then fit to data as part of training the NN. Such methods have been applied to ODEs (such as the oscillatory Lotka-Volterra system), PDEs for describing spatio-temporal biological phenomena (Rackauckas et al., 2021), and chemical master equations describing stochastic kinetics of small genetic networks including feedback loops (Jiang et al., 2021). Hence, they have proven to be very convenient when commonly used mathematical functions do not provide a model with a good fit to data. Bringing universal ordinary differential equations together with SINDy provides the opportunity, as in Rackauckas et al. (2021), to determine an unknown time-dependent reaction rate, followed by approximating the best mathematical definition of the reaction rate using SINDy. This would allow models to simultaneously be constructed directly from data whilst building on pre-existing knowledge (contained in f(x) ).

In a complementary approach, one can use the output of the NNs (e.g., a plot of NN(x) vs. x ) to estimate the precise mathematical expression (functional form) that describes an unknown term (Lagergren et al., 2020; Daryakenari et al., 2024). Lagergren et al. (2020) showed that MLPs could be used to estimate cell growth and diffusion terms in a PDE model describing scratch assay experiments where cells repopulate available space on a surface. From this analysis, explicit mathematical functions could be approximated to create a phenomenological that then showed these two terms were not sufficient for a fully accurate MLP fit. Based on this discrepancy, the authors also added a time-delay term which yielded a better model fit, even when taking into account the increased number of parameters. This methodology was demonstrated on both simulated and *in vitro* data.

### 3.3 No candidate terms are known

As a third approach, neural ODEs (nODEs) (Chen et al., 2019) can be used to estimate the rate of change of the system. Here, no underlying assumptions about the functional form of the dynamics are made, and the neural network outputs the rate of change of x ,

$$\frac{dx}{dt}=\text{NN}\left(x,t\right).$$ (7)

nODEs have been applied for transcriptomic forecasting (i.e., predicting gene expression over time) (Erbe et al., 2023), but provide limited biological interpretability. To enhance interpretability and integrate biological insights, Hossain et al. (2024) incorporated prior knowledge into the neural network architecture, specifically by adding soft constraints which steer the nODE connections to putative transcription factor-gene interactions obtained through transcription factor binding site enrichment (comparable to Section 2.1). The methodology was performed to model gene expression changes in yeast cell cycles, breast cancer progression, and B cell dynamics from ChIP-seq and RNA-Seq datasets. This approach increased performance, led to a sparser NN, and could be used to reconstruct underlying gene regulatory networks. Potentially, this gene regulatory network could be used as a starting point for a more insightful mechanistic model, built up using some of the aforementioned methods. For single-cell transcriptomics, Chen et al. (2022) and Zhang J. et al. (2023) used an autoencoder to predict RNA velocities or expressions, respectively. To gain biological insights into the workings of the autoencoder, the latent layer could be probed for biological insights.

Nevertheless, elucidating the inner workings of nODEs remains a challenge compared to more traditional ODE/PDE models. Moreover, their predictive performance can still be improved, especially for sparse, noisy biological data measurements.

### 3.4 Neural networks to enhance model simulations

Once model equations have successfully been obtained, the next step in model construction is to define parameters and simulate the system. During parameter optimisation (i.e. data fitting), a differential equation model is solved many tens of thousands of times with different sets of parameter values before the output simulations are compared with experimental data. In the absence of extensive parallelisation, the computational cost of numerically solving the model often leads to long run times for parameter optimisation. Since traditional ODE solvers are computationally demanding, researchers have considered the use of NNs to output the solution of an ODE given time t as an input. The NN is then trained to minimise a loss function that ensures the NN’s output adheres to the underlying ODE (Grossmann et al., 2023).

This approach can be extended to PDEs, providing the NN with time and spatial coordinates as has been done by Han et al. (2018), Nabian and Meidani (2019), and Wang and Wang (2024) for high-dimensional systems consisting of 50–100 equations. In these examples, the spatial coordinates of the PDE are modelled using a stochastic time-dependent processes and used as inputs into an NN to predict the evolution of system components over space and time.

Comparisons between this NN-based ODE/PDE solving method and traditional approaches, such as finite element methods, reveal two key insights (Han et al., 2018; Nabian and Meidani, 2019; Grossmann et al., 2023; Stiasny and Chatzivasileiadis, 2023; Wang and Wang, 2024). First, there is debate as to whether NNs can predict solutions to differential equations with similarly high accuracy as their finite-element counterparts. For example, Grossmann et al. (2023) show that their methodology provides PDE solutions with higher relative error compared to finite-element methods. Notably, the relative errors found in Grossmann et al. (2023) are comparable with those for high-dimensional systems (Han et al., 2018; Wang and Wang, 2024). Second, the evaluation time of differential equation systems using NNs does not change with the accuracy of solutions, in contrast to finite element methods which take longer when higher accuracy is required (Grossmann et al., 2023). This hints to the possibility that parallelisation of NN evaluation could dramatically speed up large-scale model simulations at the cost of slightly decreased accuracy of numerical approximations. To the best of our knowledge, researchers have not yet been able to bridge the gap in relative error between NN solutions and solutions obtained using finite-element methods.

In summation, the examples above illustrate how ML methods can be applied to differential equation models to identify what terms should be used in equations, predict novel terms in equations, and speed up numerical approximation of complex models.

## 4 Integrating mechanistic models and ML

### 4.1 ML to aid in fitting sparse, noisy data

Many of the methods discussed above require numerous time point measurements with minimal noise, which is often difficult to achieve for biological problems. Hence, generating an estimation of the experimental data at unmeasured time points can greatly assist in mechanistic model fitting and provide insight into the underlying biological dynamics:

$$\hat{x}=\text{NN}\left(t\right).$$ (8)

However, since MLPs commonly contain thousands of parameters, they are prone to overfitting the training data and may not generalise well to out-of-sample scenarios (Willard et al., 2022). Such function-estimating NNs can be made robust by constraining them using known ODEs, i.e., making these models physics-informed neural networks (PINNs) (Raissi et al., 2019). A first approach is to make their derivative be as close as possible to *a priori* ODE/PDEs that describe (aspects of) the known underlying biological system. Such an approach was demonstrated by Yazdani et al. (2020) on three biological datasets, and was implemented through the loss function:

$$L=\underset{\text{Data loss}}{\underbrace{{\left(\hat{x}-x\right)}^{2}}}+\underset{\text{ODE loss}}{\underbrace{{\left(\frac{d}{dt}\hat{x}-f\left(\hat{x},t\right)\right)}^{2}}}$$ (9)

The first term ensures a close match between the NN-interpolated data $\hat{x}$ and the experimental data x , while the second term keeps the MLP derivatives in agreement with the *a priori* ODEs f . $\frac{d}{dt}\hat{x}$ is found by automatic differentiation through the NN. Minimising this loss function not only allows the NN to more robustly fit the noisy training data, but also allows for simultaneous fitting of parameters in the *a priori* ODEs f . All in all, this demonstrates that the unidirectional interactions discussed so far can be integrated, where mechanistic models inform ML, and vice-versa.

On simulated datasets, Yazdani et al. (2020) demonstrate that this approach successfully estimates practically identifiable parameters (i.e., those that can be uniquely determined from experimental data) for oscillatory or adaptive models with 5–20 unknown parameters and 5–10 system variables. It would be interesting to determine how successful the methodology is with sparser experimental datasets than those used in this study. Note that this approach only works if the complete ODE equations are known *a priori*; if parts are unknown, methods as described in Section 3.2 could be used, as shown by Lagergren et al. (2020).

In this nascent field, researchers integrating NNs with biological knowledge use some ambiguous nomenclature for models, where similar methods have been given different names, and different methods have been given similar names. Table 2 provides an overview (not aiming to be complete) striving to disambiguate terminology.

**TABLE 2:** Nomenclature for integration of neural networks with biological knowledge.

Info		Characteristics				
**Study**	**Name for approach**	**Underlying ML structure**	ODE/PDE in loss function	ML-structure constrained by biological knowledge	MLP as term in ODE/PDE	ODE as input to ML (no simultaneous fitting)
Lagergren et al. (2020)	Biologically informed neural network (BINN)	MLP (fully connected) with PDE	Yes	No	Yes	No
Elmarakeby et al. (2021)	Biologically informed neural network (BINN)	MLP (sparse)	No	Yes	No	No
Hartman et al. (2023)	Biologically informed neural network (BINN)	MLP (sparse)	No	Yes	No	No
Yazdani et al. (2020)	Systems biology informed neural network (SBINN)	MLP (fully connected) with ODE	Yes	No	No	No
Przedborski et al. (2021)	Systems biology informed neural network (SBINN)	MLP (fully connected)	No	No	No	Yes
Ma et al. (2018)	*Visible neural network* (VNN)	MLP (sparse)	No	Yes	No	No
Fortelny and Bock. (2020)	Knowledge primed neural network (KPNN)	MLP (sparse)	No	Yes	No	No
Nilsson et al. (2022)	Large-scale knowledge-EMBedded Artificial Signaling-networks (LEMBAS)	RNN (sparse)	No	Yes	No	No

### 4.2 Parametrisation of metabolic systems

The use of system features alongside simulated or real data has also been applied to NNs evaluating parameters of metabolic systems, such as catalytic rates or maximal rate velocities and Michaelis constants. Choudhury et al. (2022, 2023) present REKINDLE and RENNAISANCE, that apply generative adversarial neural networks (GANs) to find sets of metabolic enzyme parameters that recapitulate metabolic profiles of *E. coli* in steady state conditions. Such mathematical models incorporate tens of state variables and hundreds of model parameters. In REKINDLE (Choudhury et al., 2022), a generator NN is trained to produce model parameter sets with such accuracy that a discriminator NN cannot predict whether they are real or fake when compared with “ground-truth” parameter sets. In RENNAISANCE (Choudhury et al., 2023), several GANs are optimised by a genetic algorithm to produce parameter sets that lead to a model consistent with experimentally determined metabolic responses (e.g., speed at which metabolic pathways reach steady state, system stability, etc.), an approach that foregoes the need for comparison with “ground-truth” parameter sets. In the initial generation of the genetic algorithm, many GANs are created and compared for their ability to produce relevant parameter sets that yield accurate steady state levels of metabolic concentrations. Following generations are then populated with GANs that are perturbed versions of the previous generations best-performing network. Over time, a population of highly performing GANs are then obtained and allows users to analyse variability of model parameters and dynamics for metabolic pathways. The output of both REKINDLE and RENAISSANCE can be used to simulate metabolic systems under different experimental conditions (at steady state or within dynamic bioreactors), compare predicted metabolic parameters with experimentally determined counterparts (and use experimentally measured parameter values to further constrain optimisation solutions), and to predict how metabolic reactions change between physiological states.

Finally, Sukys et al. (2022) have created Nessie, an NN that takes a time-point and model parameters as input and predicts probability distributions of single cell mRNA or protein copy numbers. By then comparing the distributions of system variables with experimentally-determined copy number distributions, the method allows for the back-calculation and estimation of single cell parameter distributions. The authors applied this idea to genetic feedback loops, toggle switches, and kinase pathways. The NN approach made analysis of relationships between parameters and system properties—e.g., the parameters responsible for bimodality in a simple autoregulatory feedback loop—approximately ten thousand times faster.

In summary, recent developments propose a seamless integration of NNs with mechanistic models, and we envision that further progress in this research direction will enable models with increased applicability, interpretability, and performance.

## 5 Prospective applications: from gene regulatory networks to whole organisms

In the previous sections we reviewed existing work, where mechanistic modelling constrains or informs ML methods, where ML helps construct mechanistic models, and methodologies where these two start to become intertwined. Clearly, exploiting the synergy between ML and mechanistic models can lead to more accurate, better interpretable models in systems biology, which will enhance our capacity to modify the behaviour and performance of biological systems in an informed way. Although the balance between ML and mechanistic modelling within integrated approaches may be a matter of taste, expertise of the scientist, and the availability of data and prior knowledge or models, mechanistic models in the end are most easily interpreted. In this last part we therefore turn our focus to how we envision the integration of (multiple) ML techniques could lead to the improvement and expansion of mechanistic models. Additionally, we suggest how ML methods can model residual components to improve predictive power.

### 5.1 Potential for hybrid approaches to understand tissue developmental patterning

**FIGURE 1:**
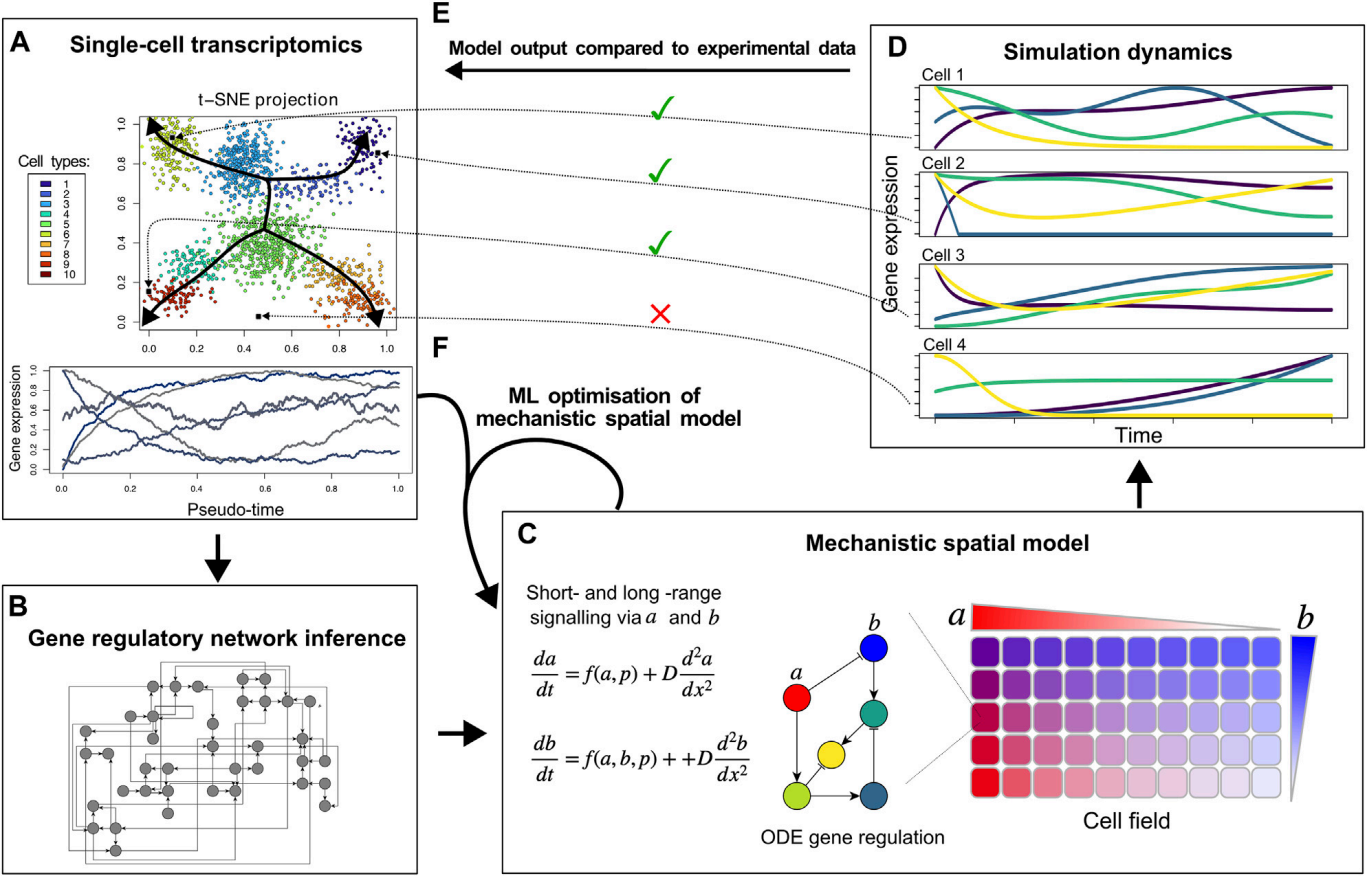
Proposed hybrid mechanistic-ML models for developmental tissue patterning. Based on single-cell transcriptomic data **(A)**, ML methods can infer a regulatory network **(B)**, that can be used as a building block of a mechanistic spatial model incorporating known and hypothesised details of cell-cell signalling and morphogen gradients **(C)**. By comparing the cell differentiation trajectories produced by the model **(D)** to the actual expression data and cell fate clusters **(E and A)**, an iterative approach can identify missing genes, short-range cell signalling, and/or morphogen gradients to optimise the hybrid model **(F)**.

As an illustrative example, in developmental biology the aim is to decipher how cells with identical genetic make up decide which genes to express when and where, in order to produce a patterned specialised tissue consisting of a variety of distinct cell types. In recent years, single-cell transcriptomics combined with ML dimensionality reduction approaches such as tSNE and UMAP (van der Maaten and Hinton, 2008; McInnes et al., 2020) are increasingly used to identify gene expression clusters corresponding to the distinct cell fates occurring in the tissue under study. Subsequently, a pseudotime-based ordering of these cell states enables the reconstruction of temporal trajectories describing cell fate development and transitions (Trapnell, 2015; Saelens et al., 2019) (Figure 1A). Thus far, these methods have mostly been used to identify novel cell types, including the gene expression profiles uniquely identifying these. Frequently, novel cell states are identified that are intermediates of previously known cell types (Jo et al., 2021; Gan et al., 2022), increasing our knowledge of the gene expression changes that cells experience on their path to differentiation. Additionally, subdivisions of previously known cell fates into distinct categories or rare novel cell types are frequently detected (Grün et al., 2015; Tang et al., 2017; Krenkel et al., 2019; Fu et al., 2020). This fine-grained level of understanding has only been possible through the combination of single-cell sequencing with ML methods.

Other ML approaches have been applied to infer gene regulatory networks from single-cell transcriptomics data, identify potential regulatory links between genes, and find the specific cell types in which these regulatory interactions take place (Aibar et al., 2017; Pratapa et al., 2020; Kamimoto et al., 2023) (Figure 1B). Still, it is highly non-trivial to determine whether the recovered regulatory interactions offer a full explanation for the observed cell fate dynamics. In fact, this may be unlikely given that single-cell sequencing is technically limited in the number of transcripts sampled for each cell, with absence of transcripts—particularly lowly-expressed transcription factors—not necessarily meaning absence of expression (Ke et al., 2023). Thus it appears an interesting research direction to combine these methods with spatially explicit mechanistic models of cell fate dynamics that can not only incorporate gene regulatory dynamics but also direct short range cell-cell signalling, longer range morphogen gradient based signalling, transcription factor complex formation, and protein stability regulation, (Figure 1C). While recently ML methods have also emerged aimed at inferring cell-cell interactions from single-cell sequencing data, this has thus far been limited to leveraging known ligand-receptor pairs (Jin et al., 2021; Wilk et al., 2023).

To construct such a mechanistic model for cell fate patterning, the regulatory network inferred by ML can serve as input into the mechanistic network model (Figures 1B,C). Likely, the ML-inferred network is large and different networks may be recovered depending on the specific inference algorithm used, potentially necessitating taking an ensemble approach (Marbach et al., 2012; De Clercq et al., 2021). Network complexity could be reduced by scoring regulatory interactions based on how frequently they are recovered by different algorithms, the integration of transcription factor binding measurements, and known transcription factor-promoter interactions. Additionally, network pruning approaches derived from NN pruning methods could be used to reduce complexity of these regulatory networks (Yeom et al., 2021).

Through simulating a mechanistic model of the multicellular tissue (cell field) that incorporates the inferred gene regulatory network, cell-cell signalling, and the role of morphogens (Figure 1C), *in silico* gene expression dynamics across the tissue can be generated (Figure 1D). Similar to the actual *in vivo* measurements, such *in silico* dynamics can be clustered into cell fates and organised according to their temporal dynamics, enabling a direct comparison with the *in vivo* data (Figure 1E). Mismatches between these simulated and actual cell fates and their dynamics can then be used to further improve and complete the mechanistic model (Figure 1F). This model optimisation should likely involve ML-based optimisation of parameters not present in the experimental data. Examples of these are protein stability, types of cell-cell signalling and their downstream effects, and/or cellular division dynamics. Finally, the integration of the mechanistic and the ML models might include the incorporation of additional relevant genes and interactions based on correlations with already modelled genes or with the phenotype aimed to be described.

Eventually, this could result in an interpretable mechanistic-ML model that reproduces ML-derived cell types, dynamics of cell fates, and inferred cell-cell signalling. We envision that iterating between model learning and adaptive weighting and pruning/sparsifying of inferred networks will help create models which balance explanatory power and model complexity.

### 5.2 Whole organism studies as a potential scenario for a hybrid mechanistic-ML model

In organisms, both local and systemic responses occur. These responses involve a wide range of spatial and temporal scales, as well as complex interactions between different organs. Here, we use plants as an example of such a multi-scale process, in which the growth and development of organs occurs throughout their lifetime and is regulated by environmental conditions like nutrient stress, drought, high temperatures, shading, or diseases. Ultimately, the organism’s performance depends on the coordination of all its parts, necessitating or the development of organism-level models that account for the dynamic processes occurring in each organ. Mechanistic models are typically limited in the number of temporal and spatial scales that can be covered within a single modelling framework, as well as in the number of relevant variables that can be considered. As an example of a modelling framework to study whole organism models, Functional Structural Plant (FSP) models integrate processes at the individual leaf and root level, overall shoot and root level, and entire plant level. In theory, FSP models can include molecular details on how each organ is regulated, e.g., root growth, even if not resolved to the level of individual cells. Still, they tend to be biased towards heavily studied adaptive responses with a clear morphological phenotype, such as preferential foraging towards high nutrient patches, stomatal closure and root elongation under drought, shoot elongation and more upright posture of leaves under high temperature and shading, and reduction in growth to redirect energy to defence under disease pressure (Ruffel et al., 2011; Huot et al., 2014; Pierik and Testerink, 2014; Quint et al., 2016; Buti et al., 2020). In contrast, transcriptomic data reveal that next to these processes with a clear observable output, a large range of metabolic and physiological responses are set in motion by stresses as well. These include changes in nitrate and carbon metabolism, membrane composition, osmotic regulation, and overall rewiring of protein translation. There are missing regulatory layers that are also important to explain an organism’s responses. The lack of detailed description of the regulation and temporal dynamics of many of these processes suggest these could be more suited for ML rather than mechanistic modelling, yet still require integration within a single model.

**FIGURE 2:**
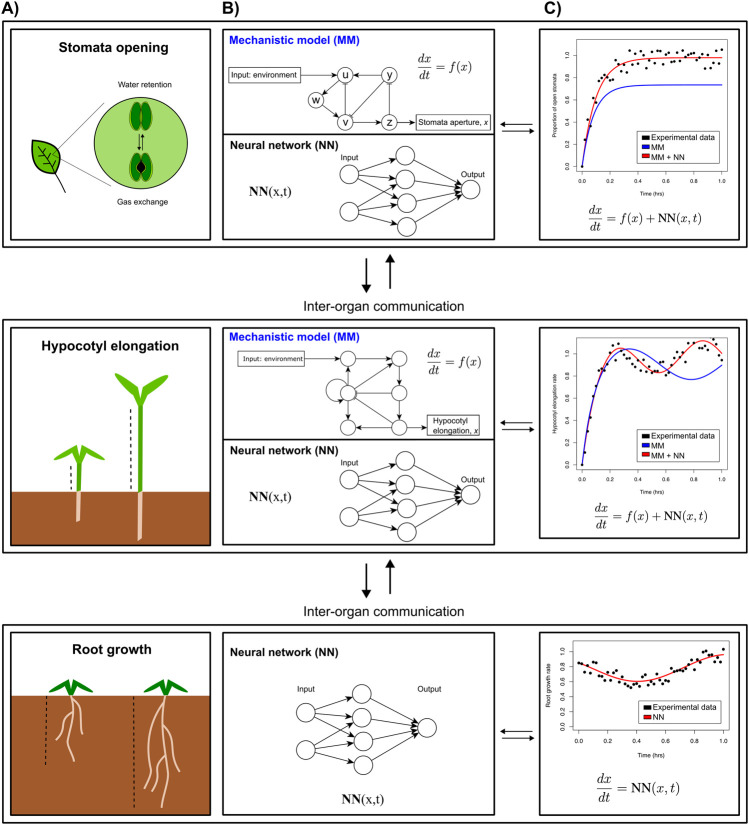
Multiscale whole organism model that models various phenotypes. **(A)**. Envisioned iterative strategy integrating mechanistic models (MMs) and neural networks (NNs) **(B)**, that in turn can be used to yield more accurate predictions **(C)**. The hybrid models developed for individual parts of an organism can then be connected to account for inter-organ communication through exchange of molecular regulators and/or nutrients.

As an example, let us assume our overall organism model contains several functional submodules governing specific morphological and physiological responses in individual organs. For a plant this will represent, e.g., root growth, hypocotyl (stem) growth, or stomatal aperture in leaves (Figure 2A). For stomatal aperture and hypocotyl elongation, key molecular players and interactions have been identified experimentally, enabling the construction of mechanistic models and explaining how they regulate plant development (Figure 2B, top part of each panel). However, many more relevant players and interactions are likely to be discovered. A promising approach to fill knowledge gaps would be to simulate these submodules using the existing mechanistic models, and compare simulated gene expression with transcriptomics measurements to determine how much of the observed dynamics of known key regulatory genes is already explained by the model, and how much “residual” is not explained yet. ML could then be used to infer which genes missing from the mechanistic model could explain these residuals (Figure 2B bottom part, c), potentially under the condition that their regulatory connections to the genes in the mechanistic model can be determined or inferred. The accuracy of the fit between the mechanistic module response and observations can then be improved by iteratively incorporating these novel genes into the mechanistic model, while ensuring high model quality measures that balance accuracy and model complexity (such as the Bayesian or Akaike information criteria, BIC and AIC). Finally, any dynamics that are still not explained by the mechanistic model—including additional genes—can be integrated through an NN term, generating a partly hybrid mechanistic ML module (Figure 2C).

A second possible application of integrated mechanistic-ML modelling would be in the many responses that are not yet properly understood or identified, but do impact the organism’s performance. Firstly, ML approaches could be developed to predict a particular phenotype, e.g., plant weight, given a number of morphological, transcriptional, and physiological responses. Feature importance assigned by the ML model would support the parametrisation of the organism-level mechanistic model. Secondly, ML approaches could be used to model the behaviour of still poorly understood response modules for which no mechanistic models can be formulated, (e.g., root growth in Figure 2B). Finally, the functional modules need to be connected (because of reciprocal dependencies or shared regulatory genes), as do different parts or organs of an organism, based on reciprocal exchange of molecular information. For plants, some root-shoot and shoot-root signals have been identified to date, yet many more likely remain to be discovered. ML-based approaches can help predict such missing connections between the different functional modules as well as distinct plant parts.

It should be noted that even though this particular section discusses plants, the foreseen approaches are equally applicable to different fields of research and other organisms, for example, in modelling a virtual human with mechanistic modules for certain well-studied organ systems, supplemented with ML modules for less well-studied parts and supported by ML-based predictors.

## 6 Conclusion

As discussed, mechanistic models are knowledge-driven approaches that offer insights into underlying biological mechanisms, but are hard to scale up to high dimensions in terms of compute time, parametrisation, and interpretability. On the other hand, ML is data-driven, allowing it to make accurate predictions using large amounts of high-dimensional data, yet it often allows for limited insight into the dynamic mechanisms underlying biological functions. Thus, the strengths of one method are the weaknesses of the other, implying that their integration would be a promising means to achieve both mechanistic understanding and accurate predictions in systems biology.

In our review, we have discussed methods which have either successfully integrated biological knowledge or mechanistic modelling into ML; used ML to help build, fit, or speed up mechanistic models; or fully integrated both approaches. Especially developments in this last category are promising; they allow each step of the procedure to be informed by its influence on the final result and help us overcome typical research challenges such as sparse and/or noisy data, unknown contributing factors, or lack of biological interpretabilty. We end with a vision on how iteratively applying several ML approaches to inform mechanistic modelling may aid in developing quantitatively detailed yet mechanistically tractable models for fields such as developmental patterning or whole organism physiology. This integrative approach promises to yield hybrid models with accurate yet biologically interpretable outputs. Such models can then be used to guide in an informed way the selection of desired behaviours of the biological system under study.

The ability to extract meaningful biological insight from SciML approaches is likely to remain a major focus for future research. Only by “opening up the black box” can we illuminate the complexities of biological processes, which are essential towards deepening our scientific understanding of mechanisms that govern the life we find all around us. Iteratively combining ML with mechanistic modelling is one of several powerful means to achieve this goal.
