# Supplementary material for: The rise of scientific machine learning: a perspective on combining mechanistic modelling with machine learning for systems biology
Source: Front Syst Biol. 2024 Aug 2;4:1407994. doi: 10.3389/fsysb.2024.1407994 (PMC12341957; doi:10.3389/fsysb.2024.1407994)
Supplement: Supplementary file 1 [file DataSheet1.PDF]

Supplementary Material

1 APPLYING SCIML IN PRACTICE

1.1 Best practices

Training machine learning models generally entails optimising some criterion (e.g. a loss function) based on example data. To investigate if machine learning models overfit—meaning they cannot generalise well to unseen data—it is considered good practice to split data up into a train, test, and validation set (e.g. 80/10/10 split). Here, the training dataset is used to train the model, and hyperparameters can be tweaked to maximise performance on the test set. After the optimal hyperparameters have been determined, the final model performance is verified on the validation set. Note that literature is not consistent on the naming of the test and validation set.

Overfitting can further be prevented through regularisation, which trades off bias (related to model complexity) and variance (related to goodness-of-fit). Such regularisation can be implemented either explicitly or implicitly. In the explicit case, one adds a regularisation term to the loss function; for example, to penalise large parameter values (also called L1 or L2 regularisation, and used in e.g. SINDY (Brunton et al., 2016)), or to penalise the ML model if it finds patterns that do not adhere to dynamics described in an existing mechanistic model (e.g. in Yazdani et al. (2020)). Implicit regularisation tackles overfitting through other means, for example through dropout layers in an NN, where parts of a layer are randomly removed during training; or through early stopping, where a model stops training if its performance on the test set starts to decrease.

Another good practice to improve model performance is to normalise (scale) input and output data, because e.g. NNs can have trouble handling data that spans several orders of magnitude (Yazdani et al., 2020). Finally, it is recommended to employ techniques like automatic differentiation in training neural networks or adjoint methods in optimizing ODEs to speed up computation. Many of the software packages mentioned below provide this functionality.

1.2 Software packages

Implementations of (some of) the SciML architectures described in the main paper are available through software packages mentioned in Table S1. Some of these packages are high-level implementations, where a large part of the underlying architecture of SciML is implemented out-of-the-box; some are low-level implementations, where models have to be built from the ground up, and only essential functionality is already provided. The former is more suitable for applying existing methods to new datasets, whereas the latter is more suitable for development of novel methods.

Table S1. Software packages to implement SciML models

Package name	Requires	Example Functionality	URL	Reference
Diffrax	Python, Jax	nODE, ODE solvers, Adjoint methods	https://docs.kidger.site/diffrax/	(Kidger, 2022)
DeepXDE	Pytorch / Jax / TensorFlow	PINN, ODE solvers	https://github.com/lululxvi/deepxde	(Lu et al., 2021)
Torchdiffeq	Pytorch	nODE, ODE solvers, Adjoint methods	https://github.com/rtqichen/torchdiffeq	(Chen, 2018)
Julia SciML	Julia	nODE, ODE solvers, UDE, PINN, SINDY, backpropagation	https://sciml.ai/	(Rackauckas et al., 2019, 2021)
Scikit-learn	Python	ML algorithms, MLP	https://scikit-learn.org/	(Pedregosa et al., 2011)
Scipy	Python	ODE solvers, parameter optimisation	https://scipy.org/	(Virtanen et al., 2020)
PySINDY	Python	SINDY	https://github.com/dynamicslab/pysindy	(de Silva et al., 2020; Kaptanoglu et al., 2022)
Pytorch	Python	Basic deep-learning functionality: e.g., MLP, RNN, LSTM, GAN, backpropagation	https://pytorch.org/	(Paszke et al., 2019)
TensorFlow	Python	Basic deep-learning functionality: e.g., MLP, RNN, LSTM, GAN, backpropagation	https://www.tensorflow.org/	(Martín Abadi et al., 2015)
Keras	JAX / TensorFlow / Pytorch	Basic deep-learning functionality: e.g., MLP, RNN, LSTM, GAN, backpropagation	https://keras.io/	(Chollet et al., 2015)
JAX	Python, Numpy	Low-level deep-learning functionality, backpropagation	https://github.com/google/jax	(Bradbury et al., 2018)

REFERENCES

Bradbury, J., Frostig, R., Hawkins, P., Johnson, M. J., Leary, C., Maclaurin, D., et al. (2018). JAX: Composable transformations of Python+NumPy programs

Brunton, S. L., Proctor, J. L., and Kutz, J. N. (2016). Discovering governing equations from data by sparse identification of nonlinear dynamical systems. *Proceedings of the National Academy of Sciences* 113, 3932–3937. doi:10.1073/pnas.1517384113

Chen, R. T. Q. (2018). Torchdiffeq

Chollet, F. et al. (2015). Keras

de Silva, B. M., Champion, K., Quade, M., Loiseau, J.-C., Kutz, J. N., and Brunton, S. L. (2020). PySINDy: A Python package for the sparse identification of nonlinear dynamical systems from data. *Journal of Open Source Software* 5, 2104. doi:10.21105/joss.02104

Kaptanoglu, A. A., de Silva, B. M., Fasel, U., Kaheman, K., Goldschmidt, A. J., Callaham, J., et al. (2022). PySINDy: A comprehensive Python package for robust sparse system identification. *Journal of Open Source Software* 7, 3994. doi:10.21105/joss.03994

Kidger, P. (2022). On Neural Differential Equations. doi:10.48550/arXiv.2202.02435

Lu, L., Meng, X., Mao, Z., and Karniadakis, G. E. (2021). DeepXDE: A Deep Learning Library for Solving Differential Equations. *SIAM Review* 63, 208–228. doi:10.1137/19M1274067

Martín Abadi, Ashish Agarwal, Paul Barham, Eugene Brevdo, Zhifeng Chen, Craig Citro, et al. (2015). TensorFlow: Large-scale machine learning on heterogeneous systems

Paszke, A., Gross, S., Massa, F., Lerer, A., Bradbury, J., Chanan, G., et al. (2019). PyTorch: An Imperative Style, High-Performance Deep Learning Library. doi:10.48550/arXiv.1912.01703

Pedregosa, F., Varoquaux, G., Gramfort, A., Michel, V., Thirion, B., Grisel, O., et al. (2011). Scikit-learn: Machine Learning in Python. *Journal of Machine Learning Research* 12, 2825–2830

Rackauckas, C., Innes, M., Ma, Y., Bettencourt, J., White, L., and Dixit, V. (2019). DiffEqFlux.jl - A Julia Library for Neural Differential Equations. doi:10.48550/arXiv.1902.02376

Rackauckas, C., Ma, Y., Martensen, J., Warner, C., Zubov, K., Supekar, R., et al. (2021). Universal Differential Equations for Scientific Machine Learning. doi:10.48550/arXiv.2001.04385

Virtanen, P., Gommers, R., Oliphant, T. E., Haberland, M., Reddy, T., Cournapeau, D., et al. (2020). SciPy 1.0: Fundamental algorithms for scientific computing in Python. *Nature Methods* 17, 261–272. doi:10.1038/s41592-019-0686-2

Yazdani, A., Lu, L., Raissi, M., and Karniadakis, G. E. (2020). Systems biology informed deep learning for inferring parameters and hidden dynamics. *PLOS Computational Biology* 16, e1007575. doi:10.1371/journal.pcbi.1007575
